# Red Cell Distribution Width is Associated with 30-day Mortality in Patients with Spontaneous Intracerebral Hemorrhage

**DOI:** 10.1007/s12028-020-01103-1

**Published:** 2020-09-21

**Authors:** João Pinho, Lénia Silva, Miguel Quintas-Neves, Leandro Marques, José Manuel Amorim, Arno Reich, Carla Ferreira

**Affiliations:** 1 Department of Neurology, University Hospital RWTH Aachen, Pauwelsstraße 30, 52074 Aachen, Germany; 2 Centro Hospitalar Universitário de São João, Porto, Portugal; 3 Neuroradiology Department, Hospital de Braga, Braga, Portugal; 4 Neurology Department, Hospital de Braga, Braga, Portugal

**Keywords:** Intracerebral hemorrhage, Red cell distribution width, Mortality

## Abstract

**Background:**

Red cell distribution width (RDW) has been associated with mortality and outcome in a wide variety of non-neurological and neurological diseases, namely in myocardial infarction and acute ischemic stroke, and the reason for this is not completely understood. We aimed to investigate RDW as a potential prognostic marker in patients with intracerebral hemorrhage (ICH).

**Methods:**

This is a retrospective study of consecutive patients with acute non-traumatic ICH admitted to a single center during a 4-year period. We reviewed individual clinical records to collect demographic and baseline information, including RDW at admission, 3-month functional status, and incidence of death during follow-up. Baseline computed tomography imaging was reviewed to classify the location of ICH, and to measure ICH volume and perihematomal edema volume. Patients were divided according to quartile distribution of RDW (RDW-Q1-4).

**Results:**

The final study population consisted of 358 patients, median age 71 years (interquartile range [IQR] 60–80), 55% were male, and median Glasgow Coma Scale was 14 (IQR 10–15), with a mean follow-up of 17.6 months. Patients with higher RDW values were older (*p* = 0.003), more frequently presented with an active malignancy (*p* = 0.005), atrial fibrillation (*p* < 0.001), intraventricular hemorrhage (*p* = 0.048), and were anticoagulated (*p* < 0.001). Three-month functional independence was similar throughout RDW quartiles. RDW-Q4 was independently associated with increased 30-day mortality (adjusted odds ratio = 3.36, 95%CI = 1.48–7.62, *p* = 0.004), but not independently associated with increased mortality after 30 days (adjusted hazards ratio = 0.71, 95%CI = 0.29–1.73, p = 0.448).

**Conclusions:**

RDW is a robust and independent predictor of 30-day mortality in non-traumatic ICH patients, and further studies to understand this association are warranted.

**Electronic supplementary material:**

The online version of this article (10.1007/s12028-020-01103-1) contains supplementary material, which is available to authorized users.

## Introduction

The global incidence of intracerebral hemorrhage (ICH), which is responsible for 10–20% of all acute strokes and associated with significant burden of disease, has increased in the past decades [1]. Mortality after ICH is significantly higher than in other forms of acute stroke, and population-based studies have shown that nearly one-third of patients with spontaneous ICH die during the first month after the event [2]. Up to this date, there are no known disease-modifying treatments; therefore, very early case-fatality has remained relatively unchanged throughout the years [3]. Most of the more robust early mortality predictors, such as age, severity of neurologic impairment, ICH volume, intraventricular hemorrhage, and infratentorial hemorrhage, are not modifiable. In the last years a great amount of attention has been paid to neuroimaging biomarkers of prognosis in ICH regarding prediction of hematoma expansion [4], but the identification of serum biomarkers also has the potential to clarify pathophysiological mechanisms and identify possible therapeutic targets [5]. Red cell distribution width (RDW) is a component of the complete blood count. It represents the coefficient of variation of circulating red blood cell volume distribution, and may reflect states of chronic systemic inflammation, poor nutrition, and microcirculation impairment [6]. Elevated RDW was found to be a prognostic marker in several vascular diseases, namely in acute myocardial infarction [7], symptomatic chronic heart failure [8], and ischemic stroke [9]. Studies have also found an association between RDW and the occurrence of delayed cerebral ischemia and poorer prognosis in patients with acute non-traumatic subarachnoid hemorrhage [10]. Up to this date, the prognostic importance of RDW in patients with spontaneous ICH is unclear, and we hypothesize that RDW could also serve as a mortality predictor after ICH.

## Aims

Our aim was to study the prognostic role of RDW in patients with acute spontaneous ICH regarding short-term and long-term survival, and to explore the relation of RDW with other well-known predictors of mortality after ICH.

## Methods

### Study Design and Patient Selection

We conducted a retrospective cohort study of all consecutive adult patients admitted to a single university hospital with the diagnosis of non-traumatic ICH during a 4-year period (January 2014–December 2017). Patients were initially selected according to International Classification of Diseases (ICD9 and 10) codes (intracerebral hemorrhage; intracranial hemorrhage, unspecified), and the clinical records of all patients were reviewed to confirm the diagnosis. We excluded patients with traumatic intracranial hemorrhage; hemorrhagic transformation of a structural parenchymal lesion (such as ischemic stroke or tumor); underlying vascular malformation; isolated subdural hematoma without trauma; isolated intraventricular hemorrhage; underlying cerebral venous thrombosis; unavailable Digital Imaging and Communications in Medicine files of first computed tomography (CT); unavailable RDW at admission. The Ethics for Health Committee of Hospital de Braga approved the study protocol and waived the need for written informed consent from individual patients (reference 153/2018).

### Data Collection

Individual clinical records were reviewed to collect demographic and clinical information at baseline and to collect follow-up information, namely functional outcome at 3 months measured by the modified Rankin Scale and occurrence of death. Early infection was defined as an infection diagnosed by the treating physician in the first 48 h after admission, treatment with antibiotics in the first 48 h after admission for presumed infection, or aural temperature ≥ 38.0° accompanied by clinical manifestations of infection in the first 48 h after admission. Isolated fever, isolated increased inflammatory markers, and use of antibiotics without clinical manifestations of infection were not considered as evidence of infection. Follow-up was conducted as part of the usual clinical care in the outpatient clinics of neurology and neurosurgery departments. Cranial CT images were reviewed to classify ICH location and the presence of intraventricular hemorrhage. ICH volume and perihematomal edema volume were measured using the manual planimetric method in ITK-SNAP [11], and raters were blinded to RDW information. ICH score was calculated for each patient [12]. RDW was measured in venous blood samples collected before CT, as part of the routine care of these patients, using fully automated measurements (Sysmex XE-5000, Sysmex Inc., Kobe, Japan).

### Statistical Analysis

The study population was categorized in four groups according to RDW quartile distribution (RDW-Q1 = 11.4–12.7%; RDW-Q2 = 12.8–13.2%; RDW-Q3 = 13.3–14.0%; RDW-Q4 = 14.1–23.1%). The groups were compared using Pearson Chi square, Kruskal–Wallis, and ANOVA tests as adequate. Spearman’s correlation was used to analyze the relationship of RDW with other continuous variables. Interobserver agreements for ICH volume and perihematomal edema volume were calculated using the interclass correlation coefficient for single measures. Kaplan–Meier curves for survival during the first 30 days and survival after 30 days for the total population and stratified according to RDW quartile were constructed, and differences between RDW quartiles were calculated using the log rank test. After visual analyses of the curves, we found a non-proportionality of death occurrence in the first 30 days after ICH (higher in the first 10 days), but not after 30 days. Therefore, univariable and multivariable binary logistic regression analyses for 30-day mortality were carried out to calculate odds ratio (OR) and 95% confidence intervals (95%CI) for the variables of interest. Variables found to be significantly associated with 30-day mortality in the univariable analysis were included in the multivariable model, except for ICH score because of overlapping variables used to calculate the score and colinearity with other variables. Likewise, univariable and multivariable Cox regression analyses were carried out for mortality after 30 days to calculate hazards ratio (HR) and 95%CI. The statistical threshold for significance was set at *p* = 0.05. Statistical analysis was performed using SPSS software (version 22, IBM, New York, USA).

## Results

**Fig. 1:**
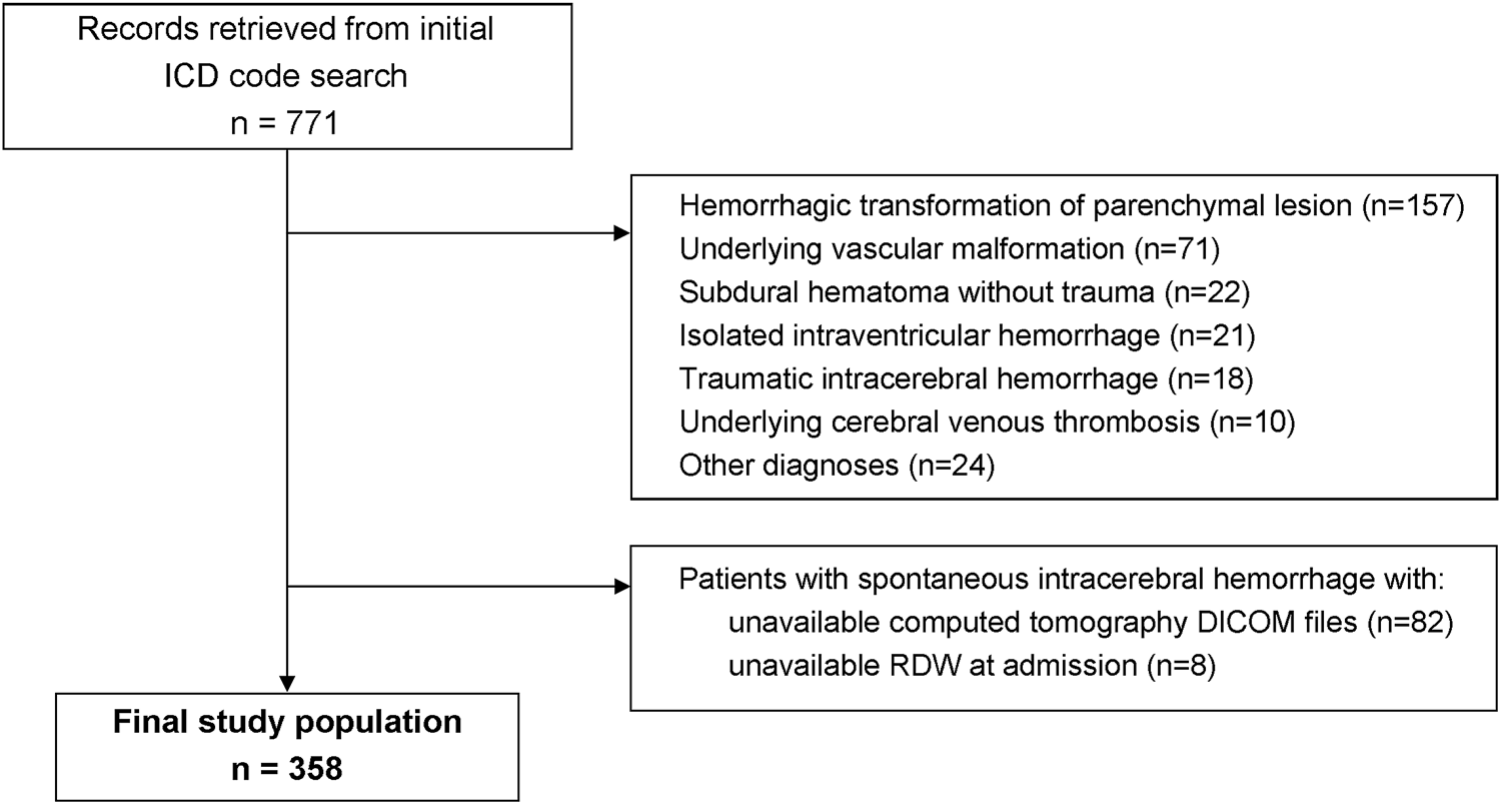
Patient selection flowchart. DICOM: Digital Imaging and Communications in Medicine. RDW: red cell distribution width

After identification of 771 records based on ICD codes, 413 patients were excluded (Fig. 1), and the final study population consisted of 358 patients. Comparisons between characteristics of patients with ICH who were excluded and patients who comprised our final study population are presented in Supplementary Table 1. The final study population had a median age of 71 years (interquartile range [IQR] 60–80), 55% were male patients, median Glasgow Coma Scale (GCS) was 14 (IQR 10–15), and the majority presented with deep ICH (54.5%). Sixty-three patients were anticoagulated at the time of the ICH, the majority of them with a vitamin K antagonist (*n* = 55) and only 8 with a direct oral anticoagulant. Most of the patients underwent CT ≤ 6 h after symptom onset (*n* = 204, 57.0%), 35 patients underwent CT > 6 h after symptom onset (9.8%), and 119 patients had undetermined time of symptom onset (33.2%). Mean total follow-up was 17.6 months (standard deviation 16.4), consisting of 520 patient/years, and 30-day mortality was 26.1%. Interobserver agreement was excellent both for ICH volume (interclass correlation coefficient = 0.98) and for perihematomal edema volume (interclass correlation coefficient = 0.94).

**Table 1:** Baseline characterization of patients according to distribution of red cell distribution width in quartiles

	RDW Q1 (n = 94)	RDW Q2 (n = 89)	RDW Q3 (n = 89)	RDW Q4 (n = 86)	p
Age (years)	66.5 (57–76)	71 (59.5–81)	70 (61.5–79.5)	75 (65–84)	0.003
Male sex	55 (58.5)	54 (60.7)	46 (51.7)	43 (50.0)	0.411
Previous functional dependency	14 (14.9)	19 (21.3)	19 (21.3)	19 (22.1)	0.577
Previous stroke	16 (17.0)	18 (20.2)	17 (19.1)	18 (20.9)	0.916
Arterial hypertension	78 (83.0)	73 (82.0)	79 (88.8)	70 (81.4)	0.525
Dyslipidemia	48 (51.1)	43 (48.3)	42 (47.2)	43 (50.0)	0.955
Diabetes	26 (27.7)	23 (25.8)	19 (21.3)	19 (22.1)	0.719
Active malignancy	0	1 (1.1)	2 (2.2)	7 (8.1)	0.005
Atrial fibrillation	7 (7.4)	9 (10.1)	19 (21.3)	25 (29.1)	<0.001
Antiplatelet therapy	30 (31.9)	29 (32.6)	22 (24.7)	18 (20.9)	0.235
Anticoagulation therapy	8 (8.5)	10 (11.2)	17 (19.1)	28 (32.6)	< 0.001
Glasgow Coma Scale, total	14 (10–15)	13 (10–15)	14 (10–15)	13 (9–14)	0.054
Systolic blood pressure (mmHg)	160 (141–182)	157 (137–180)	161 (143–182)	156 (133–179)	0.620
Diastolic blood pressure (mmHg)	88 (73–97)	82 (70–97)	83 (71–100)	89 (71–99)	0.699
Blood glucose (mg/dL)	127 (110–178)	132 (110–167)	125 (106–163)	133 (102–174)	0.768
Hemoglobin (g/dL)	14.2 (12.9–15.1)	13.6 (12.7–14.6)	13.6 (12.4-15.0)	12.8 (11.5–14.1)	< 0.001
Hematocrit (%)	41.7 (37.5–43.5)	39.8 (37.2–42.5)	40.1 (36.6–44.3)	38.3 (35.1–41.9)	0.008
Mean corpuscular volume (fl)	90.7 (87.1–94.1)	90.0 (86.9–94.2)	90.8 (86.9–94.7)	91.1 (86.4–94.3)	0.942
Platelet count (×10^3^/uL)	200 (± 56)	201 (± 55)	196 (± 68)	185 (± 77)	0.374
Neutrophil/lymphocyte count ratio	3.9 (2.2–8.2)	3.8 (2.1–6.7)	4.1 (2.0–7.3)	3.9 (2.1–7.5)	0.890
International normalized ratio	1.0 (1.0–1.1)	1.0 (1.0–1.1)	1.1 (1.0–1.3)	1.1 (1.0–2.1)	< 0.001
Activated partial thromboplastin time (s)	26.8 (24.3–29.0)	27.4 (25.5–30.4)	29.0 (25.7–34.0)	30.8 (25.9–37.7)	0.001
Prothrombin time (s)	11.9 (11.4–12.4)	12.0 (11.6–12.9)	12.4 (11.6–14.5)	13.0 (11.7–22.1)	< 0.001
C reactive protein (mg/dL)	2.9 (2.9–5.3)	2.9 (2.9–4.6)	2.9 (2.9–10.3)	4.8 (2.9–12.6)	0.001
ICH primary location					
Lobar	27 (28.7)	28 (31.5)	25 (28.1)	34 (39.5)	0.341
Deep	52 (55.3)	49 (55.1)	54 (60.7)	40 (46.5)	0.306
Infratentorial	14 (14.9)	12 (13.5)	12 (13.5)	12 (14.0)	0.992
Intraventricular hemorrhage	31 (33.0)	34 (38.2)	44 (49.4)	43 (50.0)	0.048
ICH volume (mL)	14.1 (4.8–52.2)	12.1 (4.7–29.6)	21.9 (3.9–57.9)	19.7 (5.5–37.6)	0.148
Perihematomal edema volume (mL)	12.8 (4.5–37.3)	8.9 (3.5–23.1)	15.1 (4.4–34.7)	16.8 (6.1–41.3)	0.090
Perihematomal edema/ICH Ratio	0.8 (0.6–1.1)	0.9 (0.5–1.3)	0.8 (0.5–1.1)	0.8 (0.6–1.2)	0.424
Anticoagulation reversal (among anticoagulated patients)	7 (87.5)	7 (70.0)	14 (82.4)	24 (85.7)	0.700
Early infection*	18 (19.8)	23 (27.7)	14 (16.9)	18 (22.2)	0.373
Baseline ICH score	1 (1–2)	1 (0–2)	1 (1–3)	2 (1–3)	0.030
Functional independence at 3 months†	26 (28.9)	24 (27.3)	18 (20.7)	18 (20.9)	0.461
Death at 30 days^Δ^	12 (12.8)	14 (15.7)	28 (31.8)	39 (45.9)	<0.001

ICH: intracerebral hemorrhage. RDW Q1-4: red cell distribution width quartiles

Data presented as n (%), median (interquartile-range) and mean (± SD). RDW: red cell distribution width. ICH: intracerebral hemorrhage

*Data missing for 20 patients

†Data missing for 7 patients

ǂData missing for 1 patient

^Δ^Data missing for 2 patients

Characteristics and values of clinical, laboratory, and imaging variables according to RDW quartiles are presented in Table 1. Patients in higher RDW quartiles were older, and more frequently presented with an active malignancy, atrial fibrillation and were anticoagulated. Among ten patients with active malignancy, five patients presented with hematologic malignancies and all of these presented RDW values in the highest quartile. The frequency of acute anticoagulation reversal among patients who were anticoagulated at baseline was similar in the four groups. There was a trend for patients in higher RDW quartiles to present with lower GCS scores (Spearman correlation: RDW/GCS, rho = − 0.106, *p* = 0.047), but ICH and perihematomal edema volumes were similar in the four groups (Spearman correlations: RDW/ICH volume, rho = 0.039, *p* = 0.460; RDW/perihematomal edema volume, rho = 0.055, *p* = 0.486). There was no difference in ICH location, but patients with higher RDW more frequently presented with intraventricular hemorrhage. The ICH score was higher in patients in higher RDW quartiles (Table 1), and a positive correlation between RDW and ICH score was found (Spearman correlation: RDW/ICH score, rho = 0.135, p = 0.011). Concerning laboratory variables, patients in higher RDW quartiles had lower hemoglobin and lower hematocrit values (Spearman correlations: RDW/hemoglobin, rho = − 0.231, *p* < 0.001; RDW/hematocrit rho = − 0.148, *p* = 0.005), and higher C reactive protein values (Spearman correlation: RDW/CRP, rho = 0.218, *p* < 0.001).

**Fig. 2:**
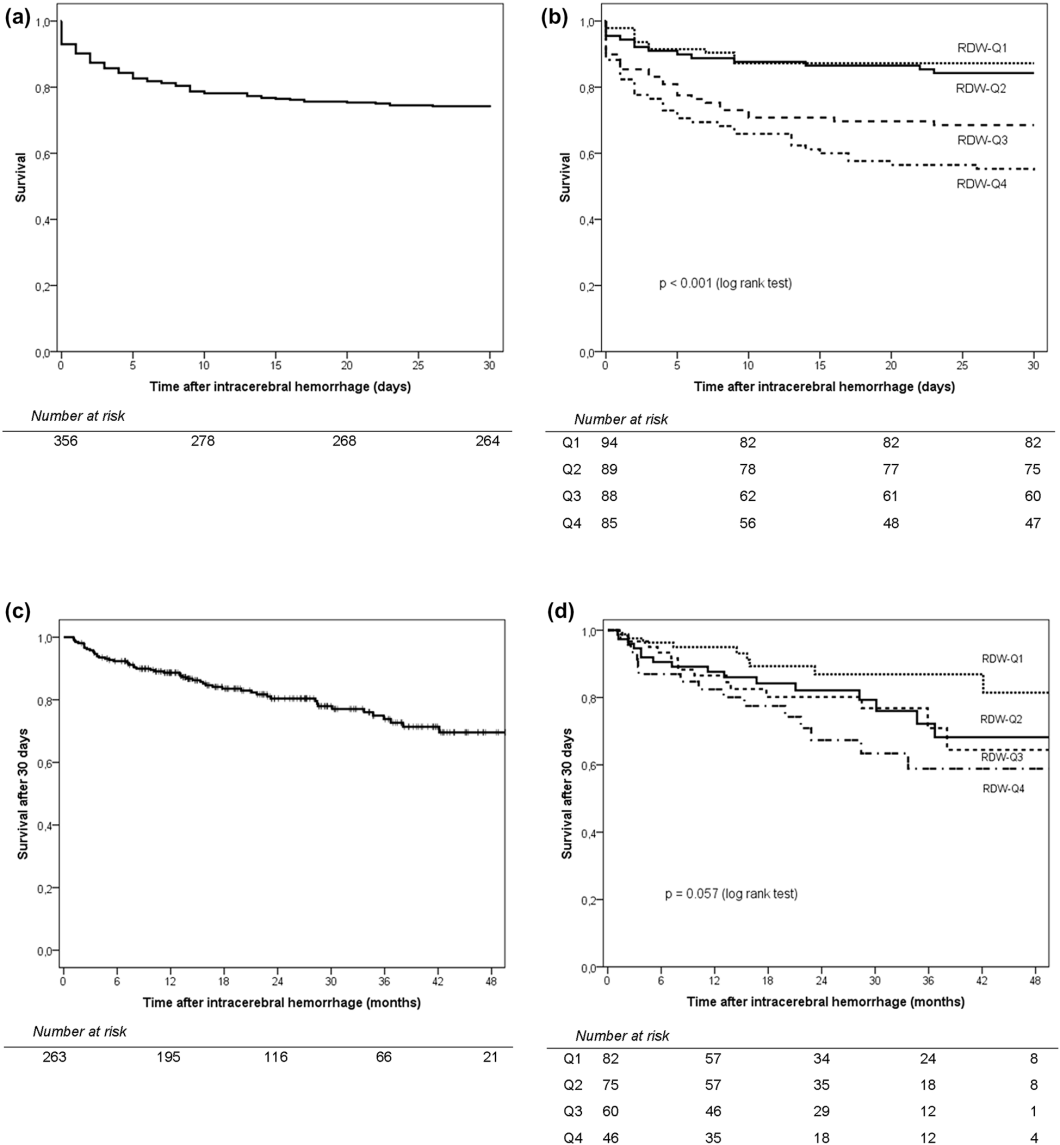
Kaplan–Meier survival curves: during the first 30 days after intracerebral hemorrhage for the total study population **a** and according to red cell distribution width (RDW) quartile distribution **b**; and after 30 days since intracerebral hemorrhage for the total study population **c** and according to RDW quartile distribution **d**

Survival curves during the first 30 days after ICH are presented in Fig. 2a, b, and show higher mortality in the groups with higher RDW quartiles (*p* < 0.001). In the univariable logistic regression analysis for 30-day mortality, belonging to RDW-Q4 group was associated with an increased risk of death (OR = 1.60, 95%CI = 1.30–1.97, *p* < 0.001). In the multivariable analysis, the only independent predictors of 30-day mortality were GCS, international normalized ratio, ICH volume, and RDW-Q4 (adjusted OR = 3.36, 95%CI = 1.48–7.62, *p* = 0.004) (Table 2). After exclusion of patients who were anticoagulated, the results of the multivariable analysis did not change significantly (data not shown). Survival curves for the period after 30 days since ICH are presented in Fig. 2c, d, and show a nonsignificant higher mortality in the groups with higher RDW quartiles (*p* = 0.057). In the univariable Cox regression analysis, belonging to RDW-Q4 was associated with an increased risk of death in the period after 30 days since ICH (HR = 1.86, 95%CI = 1.03–3.38, *p* = 0.041), but in the multivariable analysis, it was no longer a predictor of death after 30 days (adjusted HR = 0.71, 95%CI = 0.29–1.73, *p* = 0.448).

**Table 2:** Univariable and multivariable binary logistic regression analyses for 30-day mortality (data missing for 2 patients)

	Univariable analysis		Multivariable analysis	
	OR (95%CI)	*p*	OR (95%CI)	*p*
Age (per 1-year)	1.03 (1.01–1.05)	0.003	1.01 (0.98–1.03)	0.691
Male sex	0.61 (0.38–0.97)	0.038	0.75 (0.36–1.60)	0.459
Previous functional dependency	1.65 (0.94–2.89)	0.082		
Previous stroke	1.20 (0.67–2.16)	0.537		
Arterial hypertension	1.01 (0.53–1.92)	0.972		
Dyslipidemia	0.97 (0.60–1.55)	0.887		
Diabetes mellitus	1.22 (0.71–2.10)	0.464		
Active malignancy	1.93 (0.53–6.98)	0.319		
Atrial fibrillation	1.27 (0.69–2.34)	0.445		
Antiplatelet therapy	1.09 (0.65–1.84)	0.745		
Anticoagulation	1.41 (0.78–2.56)	0.258		
Glasgow Coma Scale (per 1-point)	0.72 (0.66–0.78)	< 0.001	0.78 (0.69–0.88)	< 0.001
Systolic blood pressure (per 1 mmHg)	1.00 (1.00–1.01)	0.353		
Diastolic blood pressure (per 1 mmHg)	1.01 (1.00–1.02)	0.216		
Blood glucose (per 10 mg/dl)	1.07 (1.03–1.11)	0.001	1.05 (0.99–1.11)	0.125
Hemoglobin (per 1 g/dL)	0.80 (0.70-0.92)	0.002	0.99 (0.79-1.24)	0.908
Neutrophil/lymphocyte ratio	1.04 (1.00–1.08)	0.033	0.99 (0.93–1.05)	0.632
Platelet count (per 1 × 10^3^/uL)	1.00 (1.00–1.00)	0.900		
International normalized ratio (per 1 point)	1.46 (1.11–1.92)	0.008	1.53 (1.05–2.23)	0.027
C reactive protein (per 1 mg/dl)	1.01 (1.00–1.02)	0.103		
RDW-Q4 (vs Q1-Q3)	1.60 (1.30–1.97)	< 0.001	3.36 (1.48–7.62)	0.004
Infratentorial ICH	1.57 (0.83–2.97)	0.170		
Intraventricular hemorrhage	3.40 (2.07–5.57)	< 0.001	1.37 (0.67–2.80)	0.384
ICH volume (per 1 mL)	1.03 (1.02–1.04)	< 0.001	1.03 (1.01–1.05)	< 0.001
Perihematomal edema volume (per 1 mL)	1.03 (1.02–1.04)	< 0.001	1.00 (0.98–1.02)	0.883
Perihematomal edema/ICH volume ratio	0.97 (0.79–1.19)	0.778		
Early infection	1.34 (0.74–2.42)	0.332		
Baseline ICH score (per 1 point)	2.86 (2.24–3.65)	<0.001		

ICH: intracerebral hemorrhage. OR: odds ratio. 95%CI: 95% confidence interval. RDW Q1-4: red cell distribution width quartiles

## Discussion

The major conclusion of our study is that higher RDW values are associated with 30-day mortality in non-traumatic ICH patients, and its predictive role is independent of other well-known factors associated with early mortality, namely Glasgow Coma Scale, anticoagulation, and ICH volume. To our knowledge, this is the first study to demonstrate an association of RDW with mortality in ICH patients, even though two other recent smaller studies provided uncontrolled evidence that RDW may be associated with hematoma expansion within 24 h [13, 14]. The fact that in our study, RDW was not independently associated with long-term mortality after 30 days, would suggest that it could be a marker of the severity of the initial event itself. However, even though higher RDW values were associated with increased neurologic impairment severity as measured by GCS and with increasing ICH scores, there was no correlation between RDW and ICH volume or perihematomal edema volume. We cannot exclude the possibility that RDW may be associated with other neurological and non-neurological acute life-threatening complications after ICH, and information on causes of death was not systematically available. Population-based studies have found that higher RDW values are associated with occurrence of fatal coronary events [15], of incident venous thromboembolism [16] and death related to cancer [17], but it remains to be demonstrated if they contribute to the relationship between RDW and mortality in this population of ICH patients. We also did not find an association between RDW and functional outcome at 3 months. Even though some of the well-known predictors of mortality after ICH are also predictors of functional outcome in ICH survivors [2], RDW appears not to be sensitive to short-term functional outcome. Another hypothesis for explaining this lack of association is a survivorship bias in the groups with lower RDW values, in which a higher short-term survival would consequently be associated with a relative increase in patients with more severe deficits, thus balancing the deleterious effect of RDW on functional outcome.

In accordance with the current literature, atrial fibrillation [18] and active malignancy [17] were more frequent in the groups with higher RDW values, and although they were not predictors of mortality, we cannot exclude a partial contribution to the higher mortality in higher RDW quartile groups. Another possible bias was that groups with higher RDW values were more frequently anticoagulated at baseline, which significantly increases early mortality and hematoma growth, but the predictive role of RDW was also independent of baseline INR. Likewise, the multivariable analysis for 30-day mortality included hemoglobin as a covariate, which reduces the potential bias of anemia as a relevant contributor for increased mortality.

The exact mechanisms explaining why RDW is an anemia-independent predictor of mortality for so many different pathologies, including ischemic stroke [9], subarachnoid hemorrhage [10], acute myocardial infarction [7], atrial fibrillation [18], heart failure [8], acute and chronic renal failure [19, 20], idiopathic pulmonary hypertension [21], acute pancreatitis [22], septic shock [23], cancer [24], and out-of-hospital cardiac arrest [25], remain elusive. Possible pathophysiologic mechanisms such as the association of higher RDW with reduced erythrocyte deformability and consequent microcirculation flow impairment, with nutritional deficits and with chronic systemic inflammation, and oxidative stress, have been listed by several authors [6]. A consistent and robust association of RDW with age and disease burden has been found in several studies [26], and Patel and collaborators have proposed that increasing RDW may, in fact, reflect impairment of multiple physiologic systems related to the aging process [27]. It has been suggested that increased levels of erythropoietin with aging may represent a compensatory mechanism for subclinical blood loss, decreased red blood cell lifespan, and an increased erythropoietin resistance of red cell precursors [28]. Another mechanism which could explain the association of increased RDW with senescence is reduced red blood cell survival due to excessive oxidative stress, known to occur in conditions with accelerated aging such as Down syndrome [29]. In our study, we also found that age was significantly correlated with RDW. This strong correlation and colinearity raises the question of the relative contribution of age and RDW for mortality, which may be difficult to discern [9].

The positive correlation between RDW and CRP in the absence of an association between RDW and early infection, suggests that RDW may indeed be a marker of baseline chronic inflammation. A possible link between RDW, chronic inflammation, and mortality in patients with ICH is the growth of perihematomal edema during the first days after ICH, which is known to be associated with mortality after ICH [30]. A small study in patients with ICH in whom serial magnetic resonance imaging was performed, found a delayed peak perihematomal edema volume in patients with increased hematocrit, but RDW was not reported [31]. Although we did not find an association of baseline RDW with baseline perihematomal edema, further studies should investigate whether higher RDW is associated with increased perihematomal edema growth, which is a potential therapeutic target [32].

The main limitations of our study are related to its retrospective nature, exclusion of 18% of ICH patients because of unavailable CT images for analysis, absent systematic follow-up CT for evaluation of hematoma and edema growth, and absent systematic long-term follow-up for all patients. Information on the causes of deaths was not available for our study, but could provide further understanding of the relationship between RDW and mortality. The main strengths include the fact that the study mirrors a real-life clinical setting, comprehensive clinical, laboratory and imaging characterization of the study population, relatively few missing baseline and follow-up data for the study population, and adjustment of the analyses for the more significant predictors of mortality in ICH patients.

In conclusion, RDW is a robust and independent predictor of 30-day mortality in patients with spontaneous ICH, but does not independently predict long-term mortality. Additional studies to understand the relationship of RDW with early mortality in this population of patients are warranted.

## Electronic supplementary material

Below is the link to the electronic supplementary material.

Supplementary material 1 (DOCX 12 kb)
