# Supplementary material for: Red Cell Distribution Width is Associated with 30-day Mortality in Patients with Spontaneous Intracerebral Hemorrhage
Source: Neurocrit Care. 2020 Sep 21;34(3):825–32. doi: 10.1007/s12028-020-01103-1 (PMC8179905; doi:10.1007/s12028-020-01103-1)
Supplement: Supplementary file 1 — Supplementary material 1 (DOCX 12 kb) [file 12028_2020_1103_MOESM1_ESM.docx]

SUPPLEMENTARY MATERIAL

Supplementary Table 1: Characteristics of included and excluded patients with spontaneous intracerebral hemorrhage

|  | **Included patients (n=358)** | **Excluded patients (n=90)** | **p** |
| --- | --- | --- | --- |
| Age (years) | 71 (60-80) | 66 (53-75) | <0.001 |
| Male sex | 198 (55.3) | 45 (50.0) | 0.366 |
| Arterial hypertension | 300 (83.8) | 59 (65.6) | <0.001 |
| Dyslipidemia | 176 (49.2) | 38 (42.2) | 0.239 |
| Diabetes mellitus | 87 (24.3) | 20 (22.2) | 0.679 |
| Atrial fibrillation | 60 (16.8) | 19 (21.1) | 0.333 |
| Previous stroke | 69 (19.3) | 16 (17.8) | 0.746 |
| Antiplatelet therapy | 99 (27.7) | 15 (16.7) | 0.032 |
| Anticoagulation | 63 (17.6) | 16 (17.8) | 0.968 |
| Glasgow Coma Scale, total | 14 (10-15) | 14 (10.15) | 0.997 |
| Blood glucose (mg/dl) | 130 (108-171) | 141 (113-186) | 0.140 |
| Intracerebral hemorrhage location |  |  |  |
| Lobar | 114 (31.8) | 49 (54.4) | <0.001 |
| Deep | 195 (54.5) | 17 (18.9) | <0.001 |
| Infratentorial | 50 (14.0) | 24 (26.7) | 0.004 |
| Intraventricular rupture | 152 (42.5) | 41 (45.6) | 0.596 |
| Death at 30 days^*^ | 93 (26.1) | 21 (23.3) | 0.588 |
| Independence at 3 months^†^ | 86 (24.5) | 16 (19.0) | 0.289 |

Data presented as n (%), median (interquartile range)

* data missing in 2 patients

† data missing in 13 patients
